# Lectures

**Published:** 1844-08-24

**Authors:** Benjamin Collins Brodie

**Affiliations:** Consulting-Surgeon of the Hospital


### THE MEDICAL EXAMINER,

### And Record of Medical Science.

Vol. VII.] PHILADELPHIA, SATURDAY, AUGUST 24, 1844. [No. 17.

### CLINICAL LECTURES AND REPORTS.

### LECTURES

*Delivered in the Theatre of St. George's Hospital, in the session* 1843-44,

BY SIR BENJAMIN COLLINS BRODIE,

Consulting-Surgeon of the Hospital.

*Fistula in ano. Ulceration of the intestinal mucous membrane. Cases of ulceration and perforation of the intestinal canal. Fistula in ano caused by ulceration of the rectum. Causes of the ulceration. Symptoms of abscess near the anus. Situation of the orifices. Treatment. Ward's paste. Operation.*

Gentlemen,—I presume that you are all aware of the fact that abscesses are very liable to form in the vicinity of the rectum, and that when so formed they heal only with considerable difficulty, and, for the most part, do not heal spontaneously. You are also aware that the parietes of these abscesses contract, and become hard and callous, in which stage the disease takes the name of *fistula in ano*.

Now, this affection, although of frequent occurrence in hospital practice, is much more common in private practice, and, therefore, it is, in every point of view, a disease of great interest to the surgeon.

The first question that presents itself is this,—Why is it that abscesses are so particularly liable to form in the situation in question, and that when so formed they do not heal like abscesses occurring in other parts of the cellular membrane? I formerly supposed that the healing process was prevented chiefly by the irregular action of the sphincter and levator ani muscles. Further consideration, however, and more mature experience, have lead me to the conclusion that this opinion was incorrect. That such causes may interfere with the healing of any abscess I well know, but I am now fully satisfied that they will not afford sufficient explanation why it so rarely happens that abscesses near the rectum heal spontaneously, and at any rate, it is quite clear that the action of these muscles will not explain the formation of these abscesses. In order to explain their formation I must call attention to what happens in other parts of the intestinal canal. The mucous membrane, under a variety of circumstances, is liable to ulcerate. In patients who die from diseased liver, or phthisis pulmonalis, or at the end of continued fever, and various other diseases, you find the mucous membrane of the bowels ulcerated. This ulceration seldom extends further, does not involve the muscular tunic, but sometimes the latter is affected, and then some of the contents of the intestines escape. Should this occur where the intestine is covered by peritoneum the contents may escape into the peritoneal cavity. For example, there was a little boy, seven years of age, who had symptoms of mesenteric disease, and who had just recovered from what was supposed to be a fever. When he appeared to be convalescent he was suddenly seized one evening with what was called a fainting fit, in which his pulse was not perceptible. After some time, under the influence of a stimulant, he recovered; nevertheless, he continued low and depressed. On the following day he had another attack of the same kind, from which he did not rally, and on examining the body after death I found that there was ulceration on the inner surface of the ileum, and that the mesenteric glands were diseased. In one place the ulcer had extended by a small opening through the muscular tunic, and also through the peritoneum, and a small quantity of the feculent matter had escaped into the cavity of the belly. Every person who has had much experience of disease has seen cases of the same kind; but there are others in which both the muscular tunic and the peritoneal coat ulcerate, and yet the contents of the intestine do not escape into the cavity. Adhesions take place round the ulcerated spot before the ulceration of the peritoneal coat is completed, and these adhesions cause the contents to escape, not into the peritoneal cavity, but to become infiltrated into the cellular membrane of some part of the abdominal parietes. A young man, of seventeen or eighteen years of age, who had long been in ill-health from disease of the lungs, and who was indisposed in other ways, was supposed to be rather better than usual, but one evening he was seized with violent pain in one side, and there was considerable tenderness of the whole of the abdomen. Two physicians were sent for; the symptoms were not exactly those of peritoneal inflammation, but they could not explain the symptoms so well in any other way as by assuming that he laboured under peritoneal inflammation. The inflammatory symptoms subsided, and two or three months afterwards I was called in to see him on account of a tumour which had formed in the front of the belly. It was an abscess; I opened it, and there came out pus, and with it a good deal of foreign matter, which I was satisfied must have come from the intestinal canal. The abscess made its way in several other places, and ultimately this young man died. On examining the body after death it was found that there were ulcerations at the lower part of the ileum; one of these ulcers had extended through the muscular and peritoneal tunics, but around that ulcer the ileum had contracted adhesions to the abdominal parietes above the groin, and the matter had escaped into the cellular membrane between the layers of the abdominal muscles, and from thence had made its way forward to the part where I opened it. This patient died, but it is not a matter of course that, under such circumstances, such should be the result. I was called in to see a little boy who had been supposed to labour under mesenteric disease, and I found that there was an abscess near the umbilicus, discharging pus and feculent matter. We attended to his general health, kept him in a recumbent position, lying on his back, which, I apprehend, was a most essential part of the treatment. Some very simple dressing was applied to the opening, and the boy ultimately recovered. I do not know whether he is still alive, but he was alive two or three years afterwards. I saw another case of the same kind, and I know that the boy lived a considerable time, but as he was taken away from London I cannot tell whether he ultimately recovered or not.

That part of the intestine in which ulceration of this kind is most likely to take place is the lower part of the ileum, but it not unfrequently occurs in the cæcum. Abscesses in the right iliac region generally belong to the cæcum. A young man, on jumping from a coach, felt something, as he said, give way in the right groin, and he came to London with a tumour in that situation. I thought that there was a deep-seated gland which was suppurating, and recommended him to go home, to keep quiet, and to poultice the tumour. A month after he had jumped from the coach he sent to say that the abscess had burst. There was a discharge of pus from it, but it was of a very offensive character, and on examining it carefully I was satisfied that there were fæces mixed with it. He had no bad symptoms at first, and being a very nervous man I did not tell him the exact nature of the case lest it should alarm him. Two or three days afterwards he took some medicine, and a draught of decoction of bark. A short time after, to his horror, the decoction of bark ran from the groin and frightened him out of his wits. From that moment his nervous system began to give way, he became in a state of great nervous excitement, and died ten days after the bursting of the abscess.

On a post-morten examination I found an ulcerated opening of the cæcum ; the fæces had escaped through it into the cellular membrane at the posterior part of the cæcum, and formed an abscess, which burst into the groin. There was a woman in this hospital, with an abscess in the groin, and we supposed it to be connected with dead bone, which is the case with a great number of chronic abscesses. Perhaps sufficient attention was not paid to the quality of the discharge ; but one day the woman, in taking off a poultice, found in it a lumbricus. She ultimately died, and on examining the body after death we found an ulcerated opening of the cæcum through which this intestinal worm had made its way. It was evident that the ulcerated opening of the cæcum, which was at the posterior part, had been the beginning of the abscess. Though these cases are not exceedingly rare, I mention them, because stating specific cases will often impress an important fact on the mind much more than a general observation.

I believe that this is the way in which fistulæ in ano are always formed, namely, the disease is originally an ulcer of the mucous membrane of the bowel, extending through the muscular tunic into the cellular membrane external to the intestine; and I will state my reasons for entertaining that opinion. The matter is one of great interest as a question of pathology, but it is one of great importance, as I shall show by and by, in connection with surgical practice. It is admitted by every one that in the greater number of cases of fistulæ in ano there is an inner opening to the gut as well as the outer opening; and I am satisfied that the inner opening always exists, because I scarcely ever fail to find it, now that I look for it in the proper place and seek it carefully. I have, in a dead body, examined the parts where fistulæ had existed several times, and in every instance I have found an inner opening to it. This affords a very reasonable explanation of the formation of these abscesses ; it is almost impossible to understand, on any other ground, why suppuration should take place in the vicinity of the rectum more than in any other part of the body, and why the cellular membrane there should suppurate more than cellular membrane elsewhere. Moreover, the pus contained in an abscess near the rectum scarcely ever presents the appearance of laudable pus,—it is always dirty-coloured and offensive to the smell,—sometimes highly offensive, and occasionally you find feculent matter in it quite distinct. There is no reason why an abscess simply formed in the cellular membrane should smell of sulphuretted hydrogen ; but there is a good reason why it should do so if it be connected with the rectum.

This being the case, it is easy to understand why these abscesses do not heal. The least quantity of mucus even from the gut, or of feculent matter, issuing into the cavity of the abscess, is sufficient to occasion irritation and prevent it healing, and I have more than once, in the living person, been able to trace the progress of the formation of one of these abscesses. For example, I was sent for to see a lady who complained of some irritation about the rectum, and on examining it I found an ulcer at the posterior part. I ordered her to take Ward's paste, confec. piperis nigrum, or cubebs pepper, I forget which. A month afterwards she again sent for me, and I found that there was an abscess. I opened it, and from the outer opening the probe passed into the gut through the ulcer which had been the original cause of the disease. The original opening of the abscess is generally very small indeed, but occasionally it is large, and when the ulceration has proceeded to some extent, large enough to admit the end of the little finger. The inner orifice is, I believe, always situated immediately above the sphincter muscle, just the part where the fæces are liable to be stopped, and where an ulcer is most likely to extend through both the tunics.

I believe that the most common cause of abscess of this kind is, the lodgment of hard fæces in the bowels ; by the straining that takes place to expel them the mucous membrane gets torn or abraded at one part, and then the passage of the fæces causes ulceration. Some time afterwards straining again occurs, and then the muscular tunic gives way, and the fæces escape into the cellular texture. Foreign bodies, however, in the rectum, sometimes cause abscesses. I mentioned two cases in the last lecture, but I shall mention them again. I mentioned them because they particularly appertained to the subject we were then discussing. I was called in by a gentleman who complained of great irritation about the rectum. I thought that he laboured under internal piles, but the next day he complained still more, and on examination of the rectum I found a hard substance sticking in the membrane. It was a piece of apple-core which he had swallowed the day before, and if it had not been extracted it would have occasioned ulceration, some of the fæces would have been pressed through the opening, and in all probability the apple-core would have been found in the cavity of the abscess. I was sent for to see another gentleman who was exceedingly ill with a large abscess in front of the anus. He had a brown or rather a black tongue, and bad typhoid symptoms. I opened the abscess freely, let out a quantity of putrid offensive matter, and, on introducing my finger into the abscess, I found a long fish-bone sticking across, with one end in the gut and the other in the abscess. He had swallowed the bone, it had stuck in the bowel, and a little of the fæces escaping by the side made a putrid abscess. Patients with disease of the liver, disease of the lungs, and in certain states of ill-health, are specially liable to abscess and fistula of the rectum. The reason is this: persons thus affected are peculiarly liable to ulcer of the mucous membrane; one of the mucous glandules is attacked, and being very thin it gives way under the straining that takes place to expel the fæces, and fæces escape through the opening.

The first formation of an abscess about the rectum is not in general attended with very urgent symptoms. The patient has a sense of bearing down, a fulness and weight; he thinks that he has got piles, he puts his hand by the side of the anus, and finds a little hardness. After a time it increases, the parts become tender; there is pain when he passes his evacuations; perhaps some difficulty in passing them; by and by the pain becomes still greater, the skin inflames, the abscess, if left to itself, bursts, and a quantity of matter is discharged, which matter is almost invariably offensive, dark-coloured, and putrid. The disease sometimes forms so insidiously that the patient is not cognizant of it till the abscess has burst. Twenty years ago a physician in large practice in London felt very ill, languid, listless, unfit for business; and in the middle of the day, in consequence of headach and an incapability of exertion, wanted to go home and lie down for an hour before he could finish seeing his patients. One afternoon, intending to walk home, he had sent away his carriage. He found something give way, burst into his small-clothes, and on his return he found that it was a putrid abscess—a fistula. He went through an operation for it, and got well.

While these abscesses are forming there is sometimes little or no constitutional disturbance; but in other instances there is a great deal of it, and I believe that it depends chiefly on the quality of the pus, and that, again, on the size of the opening. If the opening be pretty large, and a considerable quantity of feculent matter escapes, the pus is then of a very putrid quality, and the more putrid its character the more offensive it is to the smell, and the more poisonous it is to the patient's system; for as it is more offensive to the smell so it is more loaded with sulphuretted hydrogen. Such a collection of putrid matter sometimes produces very urgent symptoms. I was sent for to see an elderly gentleman in the neighbourhood of London with the late Dr. Blickham. I will not say that on my arrival the patient was in *articulo mortis*, but he looked as if he had not long to live—I should say hardly twenty-four hours. On inquiring into the history of the case I ascertained that he had a fistula by the side of the rectum. He had suffered under it for many years; for being afraid of an operation he had let it go on. The external orifice occasionally closed for a time, but in a few days it opened again, and gave exit to the matter. Two or three months, however, prior to the time of which I am speaking, the outer orifice had closed, and there had been no discharge, and at first no inconvenience had been felt. By and by there was a sense of pressure, a bearing down of the rectum, and the patient became very much out of health. At last typhoid symptoms supervened, and he appeared, as I have said, almost dying. I examined the parts externally, and saw that the orifice of the fistula had cicatrised. I then introduced my finger into the gut above the sphincter muscle, and I could feel an immense tumour on one side, which was evidently a large collection of matter. With the forefinger of one hand in the rectum, with the other I ran in a lancet up to the point where the matter was collected. Not only the shoulders but the whole blade of the lancet was buried before matter escaped, and then there was a little putrid discharge. With a probe-pointed bistoury I dilated the opening, and there came away a pint of such putrid matter that the whole house was poisoned by it; it could be smelt almost as bad as if a nightman had emptied his cart into it. The patient was better directly; though the incision was large there was no bleeding, and he recovered without a bad symptom. This circumstance took place many years ago, and he died lately of another complaint.

I have stated that the inner orifice of the abscess is always just above the sphincter muscle, and it may be that the abscess extends no higher than this. But in a great number of cases it does extend higher up—sometimes one inch, sometimes two; nay, I have sometimes found a probe pass four or five inches up the pelvis into a large cavity beside the rectum. These are cases of some interest, respecting which I shall have to speak to you again presently.

The external orifice of the abscess is generally in the skin, a little distance from the anus. Sometimes it seems to pass through the substance of the sphincter muscle, and on other occasions it opens externally to it. The abscess may burrow, and may be two or three inches away from the anus.

In some cases there is no external opening at all, and that may happen in two ways. I saw a gentleman who had an ulcer at the posterior part of the rectum as broad as a fourpenny piece. Some time afterwards I saw him again, and there was then a considerable discharge from the rectum, but no external opening. I introduced my finger into the rectum, and found that this broad ulcer had made a large cavity, in which matter was lodged, by the rectum. The sinus was so large that the matter had found its way out by the gut, and therefore did not burrow so as to make an external opening. But in other cases there is no external, while there are two internal openings, and they are found in the following manner:—There is a small opening through which the pus and fæces were originally infiltrated into the cellular membrane, and then the matter having collected near the gut, bursts into it, and makes a free opening in the neighbourhood of the first lesion. On examining the patient you find a discharge of pus from the inside of the rectum, and on introducing the finger you find distinctly the opening through which the abscess has burst into the rectum. This is what is commonly called blind fistula. The discharge in these cases is seldom quite constant; for the opening made by the bursting of the abscess into the rectum is not so large but what it sometimes contracts, and there not being a free discharge the matter collects, and you may feel it through the skin near the anus. This is important with regard to the treatment, as I shall explain hereafter. At other times the orifice allows the matter to escape by the rectum, and then the external tumour disappears.

In some cases there is a simple abscess and a simple sinus; but in other instances the disease is very complicated. The matter does not easily get to the surface, and it burrows in different directions; there is a sinus in this direction, and a sinus in that; sometimes it extends even to the middle of the nates, and there may be a sinus on both sides of the rectum. In these cases, where there are several sinuses, and where the disease is rendered complicated from the burrowing of the matter, it sometimes happens that there are two internal openings; but in general, however complicated the case may be, there is only one internal opening, and that communicates directly with one sinus, and indirectly with another. It is of great consequence to bear this in mind as connected with the surgical treatment. Where there are several sinuses, burrowing in different directions, the patient always experiences some degree of inconvenience. The matter lodges in one place, not in another, but wherever it lodges it occasions pain, there is an attack of shivering, and then the matter escapes. It then lodges in another place, there is another attack of shivering, and in these complicated cases the patient is continually suffering local pain and tenderness, and these are combined with constitutional disturbance.

I now come to consider the treatment of these cases. Why is it that the abscess does not heal? It may, as I supposed formerly, partly arise from the unfavourable locality for healing, in consequence of the muscular fibres of the parts being always in motion. The levator muscle and the sphincter ani are constantly drawing the parts asunder, so that they are not allowed to contract, but that is not a sufficient explanation. There is an internal opening to the abscess, and now and then a little bit of fæces or mucus will become infiltrated, and get into the cavity. That which produced the abscess originally is going on still. If you could get the inner orifice to close, the patient would soon recover. This does sometimes take place. I saw a patient who had an abscess by the side of the rectum, and to whom I recommended an operation, but for some reason or other he wished to put it off. He went about for a considerable time with this abscess, and when I saw him again the abscess was closed, and had been closed so long, and on a careful examination the parts seemed so sound, that I had no doubt that the inner orifice had healed spontaneously; the escape of feculent matter was thereby prevented, and all the parts granulated and contracted like an abscess elsewhere. The medicine which we now call confec. piperis nigri was originally a quack medicine known by the name of Ward's paste. It is composed chiefly of black pepper and elecampane, and it had the reputation of curing fistula. I believe that it sometimes did so. It is very useful in the case of piles, and where there is an ulcer of the rectum unconnected with fistula. The black pepper mixes with the fæces, it passes down the canal, and becomes a stimulant to the mucous membrane. In this point of view it is useful to persons that suffer from disease of the mucous membrane after dysentery, or who have disease of the rectum. As it will cure piles and an ulcer of the rectum, so no doubt it will sometimes cure fistula. If the little ulcerated opening can be made to contract and cicatrise there is no reason why the external abscess should not heal. But you cannot depend on such a mode of treatment as this; it may or it may not happen to cure the patient, and for one instance in which it effects a cure it fails a great number of times. The disease, however, may generally be cured by a very simple operation, and in speaking of the operation we will take the simplest case first. We will assume that there is a fistula just by the side of the sphincter muscle and only one sinus. The first thing to be done is to find the inner opening. I do not say that you will always succeed in finding it—certainly not the first time, but you will rarely fail if you look for it in the right place. Formerly, I often failed, and for this reason,—I did not know where to look for it. I used to think that it was to be found in the upper part of the sinus, but it is never found there if the sinus runs high up. You must search for it immediately above the sphincter muscle. Another circumstance that makes it difficult to find is this:—The common probe being quite round turns round in the hand; you want a probe of a much broader kind, so that the least motion of the hand turns the point another way. For this operation I use the probe I now show you, made by Philip and Wicker, in St. James's street. First, it has a flat handle, and that gives you a perfect command of the instrument; secondly, at the extremity it is like a common probe; but you must have probes of different sizes. There is a groove, so that it is both a probe and a director at the same time, and being made of silver it is perfectly pliable.

Now, to find the inner opening place the patient over a table to the light, with an assistant to hold the legs. You introduce the fourth finger of the right hand into the rectum, remembering that the opening is close to the sphincter muscle. You will feel with the finger some little irregularity, and that is where the opening is probably situated. You are then to introduce this probe into the external opening with the assistance of the finger in the rectum, using no force, and by a careful manipulation feeling first in one direction, and then in another, at last it will almost alone pass through into the rectum. It must be done gently, and a little practice will enable you to find the inner opening. You ascertain when it has passed through the opening by its coming in contact with the finger. If you do not find the opening the first day put off the operation to another day. Occasionally I have tried two or three times before I could discover the opening, but generally, if you have probes of different sizes, it is easily found. Sometimes the opening is very small, and therefore requires a small probe. When you have found the inner opening, and the probe is in contact with your finger, you bend the end, and bring it out at the anus. Thus, the part towards the handle is seen projecting from the outer opening, and the other part from the anus, while the parts which are to be divided lie upon the groove of the director. I generally divide the fistula with a pair of curved knife-edged scissors, for they cut better than a bistoury. A bistoury tears, and you may cut your own finger if you use the sharp edge. Introduce the scissors along the groove of the director, and divide the parts that lie between the inner and the outer orifice. There is scarcely anything to be divided—not above an inch or an inch and quarter, but you divide the greater part of the sphincter muscle.

Having performed this operation, all you have to do is, to prevent the cut edges growing together. You have made it into a sore, some of the fæces go into the sore, but they do not lie and lodge there, and there is nothing to prevent this fistula, which is now made into an open sore, granulating and healing. All you have to do is to dress the parts very lightly between the cut edges to prevent them growing together, and that must be continued till the cut edges are skinned over. You may then leave the parts alone, and the healing process will go on.

But suppose that the fistula extends high up by the side of the rectum, above the opening, and this fistula is burrowing, what is then to be done? I used to imagine formerly that it was necessary to lay open the whole sinus into the rectum, but it is a frightful operation to lay open so long a sinus. You do not know what vessels you divide. There is seldom much bleeding in dividing the parts, between the inner and the outer opening, but if there be much the pressure of the finger and a bit of lint stops it directly. I remember a case where I divided a fistula some way up by the side of the gut, and the whole canal was filled with blood. It is true the bleeding stopped, and the patient got well, but still he might have died from hæmorrhage. The bleeding goes on insidiously; you do not know how to stop it; it is internal, you cannot take up the vessels, and you cannot make pressure in any efficient manner. But I am now satisfied, and have been for a long time, that the division of a fistula which extends above the inner orifice is quite an unnecessary proceeding. Upwards of twenty years ago, when I was first getting into practice, I had a patient with a fistula, which I divided, or, at least, thought I had done it. But one day, when examining it with a probe, I found a sinus running up by the side of the gut for several inches. It seemed as if one side of the rectum was completely dissected from the neighbouring parts, but there was a good opening at the lower part where I had divided the fistula. Not knowing what to do with the case I called in the late Mr. Cline, and observed to him that if I divided it the whole length the patient might die from loss of blood. He said, "You are quite right, but more than that I do not think it is necessary, I would leave it alone." There was a free opening below; the fæces could not escape so easily now, and get into the cavity above. I adopted his advice, and the patient got well without any trouble. I have since seen other cases of the same kind. Where there has been a large sinus, connected with a fistula, I have laid open the parts between the inner and the outer orifice,—done nothing more,—and the patient has got well. If a very long sinus, and a very large cavity, heal up without being laid open, *à fortiori*, if there be a small sinus, and a small cavity, that will heal up to.

In the next lecture I shall call attention to the treatment of more complicated cases.—*London Lancet.*

